# Effect of Training Preparation for Childbirth on Fear of Normal Vaginal Delivery and Choosing the Type of Delivery Among Pregnant Women in Hamadan, Iran: A Randomized Controlled Trial 

**Published:** 2016-09

**Authors:** Seyedeh Zahra Masoumi, Farideh Kazemi, Khodayar Oshvandi, Mozhgan Jalali, Ali Esmaeili-Vardanjani, Hossein Rafiei

**Affiliations:** 1 Department of Midwifery, Mother & Child Care Research Center, School of Nursing and Midwifery, Hamadan University of Medical Sciences, Hamadan, Iran; 2 Department of Midwifery & Reproductive Health, School of Nursing & Midwifery, Shahid Beheshti University of Medical Sciences, Tehran, Iran; 3 Department of Medical and Surgical Nursing, Mother & Child Care Research Center, School of Nursing and Midwifery, Hamadan University of Medical Sciences, Hamadan, Iran; 4 Social Security Organization, Atiyeh Hospitals, Hamadan, Iran; 5 Chronic Diseases (Home Care) Research Center, School of Nursing and Midwifery, Hamadan University of Medical Sciences, Hamadan, Iran; 6 Department of Medical and Surgical Nursing, School of Nursing and Midwifery, Qazvin University of Medical Sciences, Qazvin, Iran

**Keywords:** Child Birth, Cesarean Section, Fear, Pregnant Women

## Abstract

**Objective:** To examine effect of an educational program on pregnant women’s fear of normal vaginal delivery. Fear of natural childbirth during pregnancy may increase the risk of caesarean section. Educational programs may be effective in reducing women fear of natural childbirth.

**Materials and methods:** This randomized controlled trial conducted from September 2012 to January 2013 in Hamadan, Iran. One hundred fifty eligible women were randomly assigned to group "A" (Intervention group, n = 75) or group "B" (Control group, n = 75). Women in group A, participated in an antenatal educations program for physiologic childbirth in 8 two-hour sessions. A self-designed questionnaire was used to examine women's fear of natural childbirth. Data were analyzed with SPSS.16 software.

**Results:** Baseline characteristics of women were similar in both groups. After intervention the mean fear score in group A compared to group B was significantly reduced (51.7 ± 22.4 vs. 58.7 ± 21.7) (p = 0.007). Physiologic delivery was the first choice of type of child birth after training in pregnant women in group A (58.7%). But delivery in physiologic form had lowest rate in group A (8%).

**Conclusion:** Results of present study showed that educational program could be serving as an important tool in reducing women fear from natural childbirth and in choosing of physiologic birth. And for delivery as a physiological, education and counseling of pregnant women, doctors and midwives are required.

## Introduction

Although cesarean section (C/S) can save mothers and offspring life in some emergency situation, but this major obstetric intervention have many minor and major short-term or long-term complications for mothers and their off springs (1). Complications for women undergoing cesarean are included bleeding, infection and embolism (2). The worldwide caesarean rate has continuously increased over recent decades either in developed or developing countries (3). The rate of caesarean section in Iran in 2010 and 2013, was 41.9% and 48% respectively (4). However, the rate of C/S that recommended by the World Health Organization for Iran in this year had been fifteen percent (5). The latest publication from the Iranian context shows that the caesarean section rate has risen in recent years in Iran (6, 7). This increase of C/S, is due to some factors such as fast economic growth, medicalization of child birth, and socio-cultural, religious and economical norms(8). One of the reasons for choosing cesarean delivery compared to vaginal delivery is fear of normal vaginal delivery among pregnant women (9, 10). This fear could be related to lack of confidence to birth, fear of the unknown, internalizing other women's negative stories, perineal tearing, labor pain, birth-related problems and procedures, attitudes of health-care personnel and sexuality were common concerns for (11, 12). In studies the prevalence of tocophobia of childbirth is different from 11% (13) to 15% (9) and nulliparous had higher level of fear than multiparous women (9). In a study conducted in Hamadan city in Iran, prevalence of fear of childbirth was 48.2% (14) among pregnant women. The pain and fear of childbirth influence the decision of women to choose the method of delivery. Because they believe that women experience less pain associated with cesarean section (15). In a meta-analysis study that was performed in Iran (2014), fear of childbirth was the first cause (39%) of cesarean delivery in this country (7). If fear and anxiety during childbirth eliminate, Mental and physical relaxation can be replaced (16). In order to achieve safe delivery and reducing the fear of childbirth and rate of cesarean delivery, physiological birth was considered. In this method labor itself begins and in a normal process progresses and common drugs and Interventions cannot be used (17). For physiological delivery, training preparation for childbirth is necessary for pregnant women during prenatal care (18). Principles of physiologic delivery and training preparation for child birth are provided according to the World Health Organization's evidence-based care (19). Childbirth preparation classes, are included group classes or individual with the aim of educating pregnant women and their spouses about labor and birth and prenatal care, nutrition and exercise during pregnancy, relaxation techniques, breathing techniques, movements situation and lactation, breastfeeding, and other cares after delivery (20). Benefits of prenatal education classes have included increased confidence for labor and birth among women who attended prenatal classes, higher likelihood of breastfeeding, improved communication between childbearing women and their maternity care providers, decreased need for analgesic medication in labor, increased satisfaction with birth (21), and correcting false beliefs about pregnancy and childbirth (22). In this study we assessed the effect of child birth preparation classes on fear of labor and type of delivery. However there have been few intervention studies that test interventions to address women’s childbirth fear (16). Present study aimed to investigate effect of an educational program on pregnant women’s fear of normal vaginal delivery.

## Materials and methods

**Figure 1:**
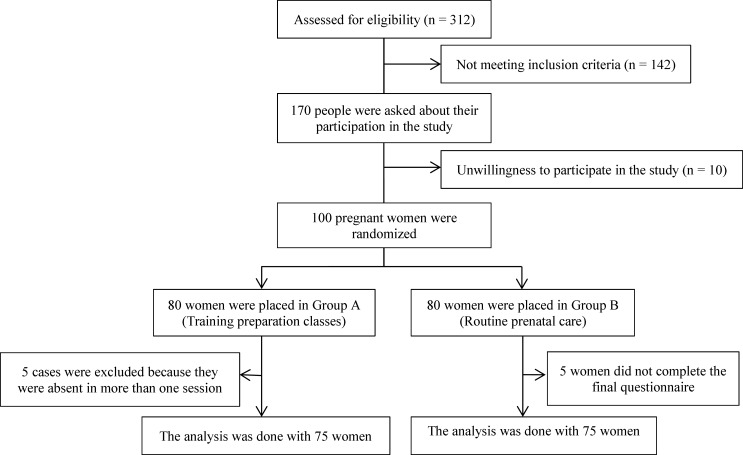
Flow of participants through the research

This randomized clinical trial study was approved by the ethics committee of the Hamadan University of Medical Sciences, and registered in the clinical trial center (reg. IRCT2014072713405N4). Study subjects were pregnant women who had been admitted to the hospital for prenatal care. One hundred and fifty women were enrolled in the study. At the first stage, 312 pregnant women were interviewed from whom 170 entered the study based on the written informed consent and the following inclusion criteria: single fetus, no chronic disease such as diabetes, heart and lung chronic diseases, no infertility, no high risk pregnancy and no history of psychiatrist visit, do not use specific drugs, gestational age of 20 weeks. They were excluded from the research in case of any problems or complications during pregnancy, failure to attend more than one session of training. Of these, 10 women refused to participate and 160 peoples entered the study and were divided into two equal groups using the table of random numbers; 80 in the intervention and 80 in the control groups. The samples were randomly selected by using of software R. All respondents gave written permission to participate in the study. Finally, 5 women in the control group did not complete the final questionnaire and in the intervention group, 5 cases were excluded because they were absent in more than one session. The analysis was conducted with 150 pregnant women (Figure 1).

For participants in group A, training preparation for childbirth and for participants in group B, routine prenatal education was done. In inside of 160 envelopes, A and B letters were written. The eligible persons were given the envelopes respectively. After opening the envelope, the type of groups was found. In addition, all participants were promised that all data would remain anonymous, kept confidential and be stored safely. Training preparation for childbirth for the intervention group was formed in 8 sessions of 2 hours. These classes were held biweekly from 20 to 34 weeks of pregnancy in Fatemieh Hospital of Hamadan City. The content of these classes were included the mother's physical and mental changes, common problems and complications of pregnancy and ways to solve them, warning signs in pregnancy, nutrition and exercise during pregnancy and lactation, training labor and delivery process, and ways of coping with them, non-pharmacological methods for pain relief and the partner’s role as a coach during labor. And for those who choose physiological delivery for method of child birth, midwife present with them during labor. And birth is done with minimal medical intervention with using of relaxation techniques and delivery ball. Every 10 to 15 people were in one group. In each session, 40 minutes were spent on practical training in breathing, relaxation, massage techniques and special exercise. Women in the control group received routine prenatal care. Before and after of training, mothers in both group completed the fear of delivery questionnaire and chose the delivery method. And the women were asked about the type of delivery after childbirth. A self-designed questionnaire was developed to examine women's fear of natural childbirth. The items were formulated based on the childbirth attitude questionnaire (CAQ) (23) and the authors experience in conducting qualitative studies in this area. The instrument was designed in Farsi language and consisted of 16 items. Answers were designed in Yes or No with minimum score of 0 and maximum 16. To facilitate the analysis the scores were converted to (0-100). Scores between (0-33.33) was considered as mild, (33.34-66.67) in moderate and (66.68-100) in severe fear of delivery. Higher score indicated more fear of natural childbirth.

**Table 1:** Frequency of low, moderate and high level of fear in women in each group

**Fear of delivery**		**Group A**		**Group B**	
		**Before intervention**	**After intervention**	**Before intervention**	**After intervention**
Levels	Low fear	7 (9.3%)	16 (21.3%)	9 (12.8%)	8 (10.7%)
	Moderate fear	38 (50.7%)	36 (48%)	36 (44.2%)	25 (33.3%)
	High fear	30 (40%)	23 (30.7%)	30 (43%)	42 (56%)

The questionnaire’s content validity was assessed by 15 members of the Hamadan Nursing and Midwifery School, who reviewed its representative and cultural aspects. These experts were also asked to rate each item based on relevance, clarity, and simplicity on a four-point scale. The researchers analyzed the results.

The content validity score was 90%. To assess the reliability of the scale, alpha coefficients of internal consistency (n = 20) were computed and was 0.92. Continuous variables were presented by mean and standard deviation. Pearson’s correlation coefficient, and paired t-test were applied for correlation and comparison. Statistical Package for the Social Sciences (SPSS 16) software was used for data analysis and p-value less than 0.05 was considered as statistically significant.

## Results

Baseline characteristics of women were similar in both groups. The mean age of women in group A was 32.9 ± 7.9 (rang from 25 to 30 years) and in group B was 32.9 ± 7.9 (rang from 25 to 30 years). Although level of education was higher in women in group A, but this difference between groups was not significant (p = 0.9). About levels of fear of childbirth, result showed that frequency of moderate (36%) and high (23%) levels of fear after intervention in group A was lower than before (38% and 30% respectively). While in group B frequency of high fear was increased (56% vs. 43% respectively) (Table 1).

After intervention the mean fear score in group A compared to group B was significantly reduced (51.7 ± 22.4 vs. 58.7 ± 21.7) (p = 0.007). And mean score in group A after training was lower than before (Table 2).

With regards to questionnaire items, women in group A obtained lower score (less fear) from many questions such as “I think that labor is an abnormal phenomenon”,“I often have nightmare about to labor”, “I'm afraid that my baby damaged during natural delivery ” after intervention. But women in group B obtained higher score (more fear) than before in most questions (Table 3).

**Table 2:** Comparison of fear score in both groups before and after training

**Fear of delivery**	**Before**		**After**		**P value**
	**M**	**SD**	**M**	**SD**	
Group A	53	19.3	51.7	22.4	t = 1.2 p = 0.24
Group B	49.1	21	58.7	21.7	t = 2.64 p = 0.01
P value	t = 0.37 p = 0.72		t = 2.75 p = 0.007		

**Table 3:** Distribution women's responses to items of questionnaire before and after intervention

**Questions**	**Group A**				**Group B**			
	**Before**		**After**		**Before**		**After**	
	**Yes (%)**	**No (%)**	**Yes (%)**	**No (%)**	**Yes (%)**	**No (%)**	**Yes (%)**	**No (%)**
Thinking to labor, affect my calmness negatively	45	55	50	50	45.7	54.3	55.7	44.3
I am afraid of being confronted with labor pain	63.7	36.3	43.8	56.2	67.1	32.9	74.3	25.7
I'm afraid to scream during labor	57.5	42.5	46.3	53.8	55.7	44.2	61.4	38.6
I always was afraid of giving birth	47.5	52.6	42.5	57.5	42.9	57.1	51.4	48.6
I think that labor is an abnormal phenomenon	13.8	86.3	8.8	91.3	12.9	87.1	22.9	77.1
I often have nightmare about to labor	33.7	66.3	28.7	71.3	30	70	44.3	55.8
I'm afraid that may vaginal tract ruptured during labor	57.4	42.6	62.4	39.6	71.4	28.6	78.6	21.5
I'm afraid of painful injections during labor	57.5	42.5	46.3	53.7	62.9	37.1	27.2	72.8
I'm afraid to loss my psychological control during labor	48.7	51.3	35.1	55	41.1	58.5	54.3	45.7
I normally prefer cesarean section to natural childbirth	65	35	27.5	72.5	31.4	68.6	44.3	55.7
I'm afraid to loss my bladder and bowel control during labor	55	45	43.8	56.2	50	50	70	30
I'm afraid that natural childbirth affect my sexual function negatively	43.7	56.3	28.7	71.3	44.3	55.7	47.1	52.9
I'm afraid of loneliness during labor	51.3	48.7	47.5	52.5	64.3	35.7	57.1	42.9
I'm afraid of hunger and thirst during labor	85	15	17.5	82.5	17.1	82.9	22.9	77.1
I'm afraid that my baby damaged during natural delivery	81.3	18.8	22.5	77.5	70	30	81.4	18.6
Natural delivery has many problems such as low back pain and etc.	72.5	27.5	63.7	36.3	65.7	34.3	67.1	32.9

**Table 4:** Comparison selection the type of delivery between two groups

**Type of delivery**	**Group A**		**Group B**		**p value**
	**Before**	**After**	**Before**	**After**	
	**No (%)**	**No (%)**	**No (%)**	**No (%)**	
Normal Vaginal Delivery	10(13.3)	24(32)	26(34.7)	15(20)	< 0.001
Cesarean	60(80)	7(9.3)	49(65.3)	59(78.7)	
				
Physiologic	5(6.7)	44(58.7)	0(0)	1(1.3)	
Total	75(100)	75(100)	75(100)	75(100)	
P value	< 0.001				

Physiologic delivery was the first choice of type of child birth after training in pregnant women in group A (58.7%). Next choices were natural vaginal birth and cesarean delivery respectively. Whereas 1.3% of women in group B had been chosen physiologic delivery. And in this group cesarean and then natural vaginal delivery were next options (Table 4).

Finally naturally child birth had the highest rate between the two groups (48%, 57.3% respectively). But physiologic delivery had lowest rate in both groups (6%, 0%). While the cesarean rate was still high in two groups (Table 5).

## Discussion

Results of present study showed that many pregnant women are afraid of labor. And one of the reasons for this fear is lack of information about the process of labor and fear of damage to the fetus. Similar to finding of present study, most previous studies showed that pregnant women have some level of fear from natural delivery (24). In some studies, researchers found that ‘process of labor and childbirth' was the most important source of fear or delivery (14, 25). Safety of baby and her mother, lack of control over unavoidable circumstances, inappropriate behavior of maternity ward staff, Fear of motherhood, Fear of tolerance of labor were the next reasons of child birth fear (26, 27). Results of Hall et al. study also showed that pregnant women's fear of childbirth was related to fatigue, available help, stressors, and anxiety (24). In our study training preparation for child birth significantly decreased women fear from natural childbirth. The main cause of the escalation of labor pain and prolonged second stage of labor, is anxiety and fear in labor. Women with attending in fitness classes and training before birth, get the opportunity to reforming the false beliefs and misinformation about pregnancy and labor and have better compliance with the various stages of labor (28). In two studies had been used psycho-education with telephone counseling intervention by trained midwives for reducing child birth fear in pregnant women. The intervention reduced fear of delivery and increased rate of vaginal delivery (29, 30). In our study after intervention physiologic delivery was the first selection in women for the method of child birth. One of the reasons of increasing the rate of C/S is fear of natural childbirth (31). In another study role play education in primiparous women reduced fear of child birth and was more in making decision on selection of normal vaginal delivery (32). In our study normal vaginal delivery was high, but physiologic delivery was still low and C/S was high. Fabian and colleagues reported that although there was no significant difference in type of delivery between Participants in the childbirth classes and non-participants, but the women in classes tended to had an epidural for pain relief in labor and delivery (33). 

**Table5:** Comparison the type of delivery between two groups

**Type of delivery**	**Group A**		**Group B**		**p value**
	**No**	**percent**	**No**	**percent**	
Physiologic	6	8	0	0	< 0.001
Normal vaginal delivery	36	48	43	57.3	
Cesarean Elective	8	10.7	8	10.7	
Cesarean Emergency	25	33.3	24	32	
Total	75	100	75	100	
P value	< 0.001		< 0.001		

Saisto in a study about the effect of group therapy in reducing the fear of childbirth expressed that 85% of pregnant women after the intervention were not willing to do C/S (34). The reasons of high rate of C/S in our study can include: mother and doctor willing to perform cesarean delivery because of fear and reducing risk appetite, views obstetricians and midwives that a significant number of obstetrics team preferred cesarean delivery method and lack of adequate training of health care workers in this regard. This study set out to examine effect of an educational program on fear of pain among Iranian pregnant women. Results showed that educational program may serve as an important tool in reducing women's fear from natural childbirth. Conducting a randomized controlled trial to compare the effect of different types of prenatal education programs on women fear of childbirth is recommended for future studies.

***Limitation***

Use of the self-reported questionnaires may have led to an overestimation of some of the findings due to the variance of different methods.
